# Housing Needs of Ageing Veterans Who Have Experienced Limb Loss

**DOI:** 10.3390/ijerph17051791

**Published:** 2020-03-10

**Authors:** Gemma Wilson, Gill McGill, Alison Osborne, Matthew D. Kiernan

**Affiliations:** Faculty of Health and Life Sciences, Department of Nursing, Midwifery and Health, Northern Hub for Veterans and Military Families’ Research, Northumbria University, Newcastle NE7 7XA, UK; gill.mcgill@northumbria.ac.uk (G.M.); alison.osborne2@northumbria.ac.uk (A.O.); matt.kiernan@northumbria.ac.uk (M.D.K.)

**Keywords:** housing, home adaptations, military veteran, older adult, healthy ageing, maintaining independence

## Abstract

Military veterans can experience limb loss as a direct result of conflict, an accident, illness or injury. Whatever the cause, there is a need to recognise the long-term consequences and challenges of limb loss on maintaining independence in one’s home. This study aimed to examine the housing needs of veterans experiencing limb loss, and the impact of limb loss on housing needs and home adaptations of ageing military veterans. Thirty-two military veterans (aged 43–95) participated in this study and up to three life-story interviews were carried out with each participant. Two themes were generated: availability of support and changing housing needs. It is evident from the findings that military veterans are unique in various ways, specifically due to military culture, geographical relocation and the additional support that is available to the Armed Forces Community. This must be considered in long-term support to maintain independence in the home.

## 1. Introduction

World War One left the United Kingdom with a legacy of over 41,000 Armed Forces personnel requiring major limb amputation. A further 9000 experienced limb loss as a result of World War Two, and recent military operations have added to the population of service personnel who have experienced limb loss. Military amputees generally include both victims of actual military conflict and of non-combatant trauma [1]. The impact of losing a limb extends well beyond initial recovery and rehabilitation, with long-term consequences and challenges requiring health and social care commitments across the life-course [2]. Existing literature highlights that veterans who have experienced limb loss are, for the most part, able to achieve and maintain good quality of life [3]. Nevertheless, the amputation of a limb, or limbs, is a progressive injury. It involves enduring experiences of pain, co-morbidities and, sometimes, mental health problems. 

There is some debate with regards to how unique military veterans are in comparison with those citizens that have lost limbs due to industrial or road traffic accidents. In the United Kingdom there is a distinct difference which is enshrined in government policy, i.e., the Armed Forces Covenant and its direction to the National Health Service (NHS), with regards to how Armed Forces personnel and veterans should be treated. Most notably the policy states, ‘that special consideration is appropriate in some cases, especially for those who have given the most such as the injured or the bereaved’ [4]. This is further enshrined in NHS England’s implementation of that policy with clear direction being given when commissioning services [4]. 

Limb loss within the Armed Forces Community has often occurred traumatically and suddenly. As a result, the cost of health and social care provision for veterans who have experienced limb loss is necessarily higher, as this group may need additional support compared to their peers. In most cases, at the point of injury, these individuals will be dislocated from their immediate family by geographical distance. Furthermore, their limb loss may ultimately lead to the total loss of military employment and career, the loss of their home (military accommodation) and a geographical relocation for post-military settlement. This invariably leads to individuals moving through multiple hospital and care providers as the transition from the military occurs, resulting in a fractured care pathway. It was as a direct result of these experiences and concerns that the Murrison Review into military amputees was undertaken [5]. The review made key recommendations that not only benefited military amputees but also civilian amputees. The most notable key recommendations included the specialist commissioning of prosthetics and rehabilitation through five centres in England to cater for those veterans leaving the Armed Forces. Furthermore, recommendations specify that veterans should be able to access mainstream NHS provision through a Disablement Services Centre (DSC) of their choice, and there should be a programme of military/civilian exchange for healthcare professionals to grow the specialist prosthetic and rehabilitation network rapidly [5]. Over the subsequent years since 2011, the Murrison Report has led to the establishment of five regional Murrison Centres, Nine Disablement Service Centres, and the Veterans Trauma Network, which provide enhanced services for wounded, injured or sick veterans. This comprehensive network of veteran-specific services cater for the unique health and social care challengers that military amputees face during their recovery, rehabilitation and throughout the rest of their lives [6]. 

The home has long been recognised as one of the main settings to influence both health and well-being [7], and suitable housing and appropriate home adaptations are essential in order to promote independence and maintain a good quality of life amongst those affected by limb loss [8]. The experience of transition from a hospital setting back into the home during early recovery from amputation may be a significant challenge for amputees and their families, and individuals must then learn how to navigate their own home. The Care Act (2014) reinforced statutory obligation, explicitly outlining the importance of suitable living arrangements alongside appropriate home adaptations [9]. In England and Wales, funding in the form of a Disabled Facilities Grant (DFG) may be provided to help an individual modify their home, and is based on a needs assessment, most often carried out by an Occupational Therapist or a social worker [10]. Home modification funding is also available in Scotland and Northern Ireland, although different to the DFG. Although adapting one’s home can be a cost-effective way to maintain independence and prevent falls and injuries [11,12,13], mainstream housing is often unsuitable for adapting to meet accessibility needs [12,13,14]. 

Even without an injury, transition from the military back to civilian life and relocation can be challenging. Invariably military personnel will always relocate geographically on leaving the Armed Forces. Service leavers must register with a civilian General Practitioner, living in a civilian local authority, and have access to services they have not accessed before. In 2015, Simpson and Leach reported that only 8% of UK veterans were correctly registered with a General Practitioner or Family Doctor practice [15]. This low figure is despite a NHS website informing veterans of the healthcare benefits and there are no perceived barriers that specifically prevent veterans from registering. Two years later, and despite significant investment in veterans’ health and social care, a recent study indicated little change. However, in a cost effective six-week intervention this was increased to 26% [16]. 

It is therefore argued that veterans transitioning from military service following limb loss are a unique group and there is a dearth of research examining the impact of limb loss on their relocation, housing needs and how to ensure long-term independence in their home throughout the life span. This study aims to address that gap, by examining the housing needs of veterans experiencing limb loss, and the impact of limb loss on housing needs of ageing military veterans.

## 2. Materials and Methods

### 2.1. Design

In order to explore the housing needs and home adaptations of older veterans who have experienced limb loss, a qualitative approach was employed to explore the experiences of ageing military veterans. The findings in this paper were extracted from a project looking at maintaining independence, specifically focusing on the health and social well-being of older veterans who have experienced limb loss [2]. 

### 2.2. Participants

Utilising peer-recruiters, 32 veterans who had experienced limb loss were recruited throughout the UK via Blesma, the Limbless Veterans Charity. Participants were aged between 43 and 95 (mean = 69.4, SD = 14.56), 30 were male and two were female. All services of the UK Armed Forces were represented: Royal Navy and Royal Marines (*n* = 7), British Army (*n* = 19) and Royal Air Force (*n* = 5); one participant was a Cadet. 

Of the 32 participants, 59.4% (*n* = 19) had experienced operational deployment during the course of their military service, while 40.6% (*n* = 13) were never deployed. Those with deployment experience served in a range of conflicts, including World War II, Suez Crisis, Northern Ireland, Falklands and the Gulf War. The mechanism and nature of participants’ limb loss was also recorded (see Table 1).

### 2.3. Data Collection

Before participants agreed to take part in the study, peer-recruiters disseminated study information and answered questions about potential involvement in the study. Because the peer-recruiters were members of the charity Blesma, they brought with them a shared understanding, and they shared experiences with potential participants. If participants wished to be part of this study, they were asked to sign a written consent form. At a later date, a member of the research team visited the participants to carry out the interviews.

Face-to-face, semi-structured life-story interviews were conducted in the participants’ own homes (with the exception of one participant who was interviewed via email on request). The semi-structured interview provided a framework to explore the physical, psychological and social well-being of veterans who experienced limb loss across the life-course. From each interview, researchers were able to ascertain factors that contributed to the ability of veterans who experienced limb loss to maintain their independence. In order to allow sufficient time for participants to share their respective full life story, up to three interviews were carried out per participant. Each interview lasted between 90 and 180 min and was recorded using a Dictaphone.

### 2.4. Data Analysis

Interviews were transcribed and entered in NVivo 12 for qualitative analysis. Thematic Analysis was used to analyse the semi-structured interview data. The six steps of Braun and Clarke [17] were followed: familiarisation with the data, generation of initial codes, searching for themes, reviewing themes, defining and naming themes and producing the report.

This study was granted ethical approval through Northumbria University’s Ethical Approval System.

## 3. Results

Two overarching themes were generated from the data: availability of support and changing housing needs (Table 2). Both themes consist of multiple sub-themes.

### 3.1. Theme 1: Availability of Support

Whether limb loss occurred during or after service, individuals often struggled to navigate sources of financial support for their housing needs or home adaptations, as they were unsure if this was the responsibility of the military, statutory services or other. In many cases, individuals relied upon military charities to support them. However, there was also a level of stoicism that affected the support they received due to the reluctance to ask for help.

#### 3.1.1. Navigating Sources of Support

As military veterans, participants were often unsure of who would ‘take responsibility’ for them and meet their needs. Participants were often uncertain if this responsibility lay with the Ministry of Defence, military charities or statutory services.

*“You’re in the military so who’s going to pay for me? Who’s actually going to take responsibility and say ‘We’re going to look after you?’”*

*(P005)*

*“I don’t know what the options [for financial help with adaptations of home maintenance] are.”*

*(P008)*

For Participant 002, there were inconsistencies in the support received from the military, and it was felt that support was inequitable. This participant felt disadvantaged because limb loss was not related to active service.

*“I don’t want just money, I… you know I’m after help and support in helping to move my life forward. Where is it? Its non-existent. Unless it… unless because of what happened to me and being so… being a peacetime injury, it’s… it has no bearing, it’s not important compared to those that are hurt through conflict.”*

*(P002)*

Participants described being misinformed or having received no information with regards to the support they were eligible for. This often meant that they missed out on financial support to which they were entitled.

*“A rep [from a military charity], he got me up here. And he was great, but as soon as I got up here and he was giving me the wrong advice. I said ‘How do I get reinstated to get my benefit back up?’ Because they’d cut it. He said ‘Oh you’ve got to go and see your doctor?’ So I made an appointment and went to see the doctor, the doctor said ‘No you’re not supposed to be coming to see me, you’re supposed to write a letter and then they send you to see me’. So he cost me three weeks and he was supposed to be my welfare officer.”*

*(P008)*

*“I told you I come out of hospital and they… no one tells you about benefits and what you can get […] But it would help you know. Because I didn’t know about disability living allowance until six months after I could have claimed it.”*

*(P023)*

Barriers did not end when individuals found the correct source of support they were entitled to, and there were also issues with ‘entitlement’ as individual need did not meet specified criteria.

*“I’ve asked for help and they’ve said basically this ‘Unless you need… can you wash yourself?’, ‘Can you dress yourself?,’ ‘Yes’, ‘Can you cook a meal?’, ‘Yes’, (umm) and ‘Can you take yourself to the toilet? Well if you can do all them four things…”*

*(P008)*

*“I was in a bungalow that I could only use the back door. I couldn’t get out the front door because there was a series of steps so I could only use one. They wouldn’t adapt it for me, the housing association refused to adapt it.”*

*(P008)*

For Participant 008, the issues arose when they were living in privately owned accommodation as opposed to housing owned by the local authority.

*“Private houses, but not council, private… yeah they didn’t want to pay to have it adapted.”*

*(P008)*

Participants had often lived abroad for long periods of time whilst in the military and this added an additional layer of complexity which impacted ‘eligibility’ checks for new housing.

*“I went to buy my house out in [PLACE] and to get a mortgage you had to put down your last three years of where you were living. So the year previous I was in [PLACE] so I had an address, two years prior to that I was [ABROAD]. So I said it was [PO Box]. ‘Oh what about utility bills?’ ‘Never had a utility bill, came out of my wages’ ‘What about a phone?’ I said ‘No didn’t have a phone. No didn’t have a phone’. ‘Well how can you prove you lived there?’ […] I gave it to them and they wrote back to the mortgage company saying ‘Never heard of this fella!’ So I had to live in the house for a further two years so I could tell the mortgage company where I lived for the prior three!”*

*(P013)*

Some participants paid for their own home adaptations due to ‘desperation’.

*“I had to pay for stair lift myself.”*

*(P020)*

*“We even brought a ramp, you know (umm) for to get the wheelchair up and down. (umm) We then had to get the door widened and a low threshold. (umm) Nothing from the… what do you call? Social service or whatever, no help at all, no. We paid for it because we were desperate. I’d spent weeks trapped in that lounge, I couldn’t get out of the lounge because I couldn’t get to the kitchen because it was a step down and to get to the toilet was… was down.”*

*(P021)*

#### 3.1.2. Support from Military Charities

Military veterans are entitled to access financial support and received financial support in the form of compensation or their pension.

*“I can’t imagine and not having the financial assistance that pensions and compensation have given me as a person with all these other problems with the cancer, limb loss and all this… and then two young children… I haven’t had a financial worry (umm) you know the kids went to private school, (umm) I can have my own hobbies, the house is paid for, (umm) and you know I don’t deliberately save all my money up and you know so I live well. And you know I’m very grateful to having the system that we’ve got and it is a welfare system isn’t it? Army pension, war pension and then criminal compensation.”*

*(P005)*

Many participants are not used to navigating the system for support to get housing adaptations because of the culture of the military; everything is done for them, and they do not utilise civilian support services. This is in contrast to civilians who have maintained their own home throughout their lives—either by owning or renting their own home. Individuals often struggled to identify non-military support services, and relied on military contacts.

*“Thanks to Blesma I… I’m able to apply for a grant to help me with my gardening and Blesma’s my… they’ve given me each year and it helps me to help myself and my partner to have some sort of work done in our garden. (umm) But I’ll be brutally honest, that’s the only financial help I get.”*

*(P002)*

*“We didn’t know how to go about getting the council to do the extension and all the rest of it. So yeah [military charity 1 and military charity 2] helped.”*

*(P010)*

*“No-one tells you about benefits and what you can get. We’ve had … [military charity 1] has been really good and [military charity 2].”*

*(P016)*

Many of the participants praised various military charities for providing funding for housing adaptations (either in the form of a loan or grant), general support with housing and garden maintenance and navigating the system between military support and statutory services.

*“They gave me a grant to get my (umm) stairs and landing decorated because I… again I could do it, but I’d probably fall of the ladder and… look if there are any specific needs like adaptions to house, don’t always provide them themselves. I think they’ll go through local authorities, but if I needed anything like that I’d probably… maybe phone up [Bill] who’s my (umm) welfare officer, have a quick chat or email him usually saying ‘Right’ you know if I need ‘X, Y, Z’ like… a grant I had in my bathroom which I’m going to.”*

*(P006)*

*“[Military charity] have been brilliant to me. Absolutely brilliant.”*

*(P016)*

*“[Military charity]—they’ve widened the doors, they’ve done the wet room.”*

*(P027)*

In some instances, this financial support was provided by a combination of organisations—both military charities and statutory services.

*“So they got the [local authority, military charity 1 and military charity 2] and I think someone else all put some money in to have an extension with a wet room.”*

*(P010)*

#### 3.1.3. Stoicism

Stoicism was one of the barriers to accepting financial or practical support from any military or statutory organisation. Participants described having a ‘get on with it’ attitude and were reluctant to receive any help. For Participant 005, it was important to consider what help was most needed, rather than asking for multiple forms of support.

*“As long as you’re not too proud to not ask for assistance. And I think there is something in that, I’m a [military position], I am so independent, it is difficult to actually go to them and actually say (umm) ‘In need of help’ And what is more vital? Practical help, financial help, welfare assistance, you’ve got to just (umm) accept…”*

*(P005)*

Like Participant 005, others discussed the importance of adjusting their own attitudes in asking for help to ensure they were able to receive support from others.

*“Accepting help where it’s needed. I’m more inclined to accept a little bit of help these days than I previously was. But (umm) and asking for help it… as well you think you know something you can’t do on your own.”*

*(P012)*

*“Doing stuff for myself, only asking for help when I really need… well I know I need something (umm) I’ll ask. But that’s just a normal thing isn’t it so (umm) yeah independence is massively important. You know don’t ask people for money and all this sort of stuff so (umm) and… be self-supporting.”*

*(P006)*

This sense of stoicism was often felt to be directly associated with being in the military and translated into their attitude to adapting and coping with their injuries. Whilst this coping mechanism promoted individual resilience, it often led to individuals not asking for help from others.

*“I’ve lost my leg and I’ve got to get on with it and you’ve got no choice!”*

*(P004)*

*“The military coping at the time was don’t be a cry-baby, just go out, get [drunk] and get over with it […] get over it, soldier on.”*

*(P001)*

*“If I was a civilian I don’t know if I would have coped. Being a military, it was let’s just move on and carry on.”*

*(P005)*

### 3.2. Theme 2: Changing Housing Needs

Participants’ housing needs changed suddenly due to the nature of their limb loss. This suddenness affected their responses to their own home and adjusted housing needs, including home adaptations. Participants were often living in unsuitable housing for long periods of time and in many cases had to adapt their own behaviour and ways of living in the absence of suitable home adaptations. The complexities in daily living were heightened by the inconsistencies with their prosthesis; sometimes participants were more mobile than others who experienced complications with their prosthesis.

#### 3.2.1. Unsuitable Housing

For many participants, their homes became unsuitable post limb loss, and many could no longer function within their home. Participant 008 describes the difficulty in maintaining his own home.

*“It takes me all day just to keep this house going.”*

*(P008)*

Participants could often no longer function in the same way as they had previously. Participant 002 gives the example of cooking. As they could no longer cook food for their family, the participant now relied upon takeaway food deliveries.

*“It was my responsibility to sort out food and I would end up having to phone takeaways and the takeaways got to know me so well that they would even just knock on the door and let themselves in and bring my food for me.”*

*(P002)*

Other participants relied upon housing adaptations and many experienced difficulties without these modifications, particularly those helping them to get up and down stairs, making the upper level of the house inaccessible.

*“My wife at the time, she (umm) decided that my bed was going to be safer downstairs and that’s where I slept for… since having the total knee replacement and then the amputation. The settee was my bed.”*

*(P002)*

*“I was living in the front room had a blow up bed, a double with me and my wife. She came down with me and I was having to have strip washes in a bowl of water for me, strip washes or clean my teeth and that and obviously couldn’t get upstairs to the toilet or through to the other one.”*

*(P010)*

*“[Before having a stair lift] I used to go upstairs… I had to go upstairs on my back… on my bum.”*

*(P033)*

Another issue relating to unsuitable housing was the lack of external accessibility to enter and exit, meaning that many felt stuck in their own homes.

*“I am bored being stuck at home and it allows me to ponder and think long and hard about this and it does make me angry and it does get me upset.”*

*(P002)*

*“I used to arrive home and I used to have to stay in the house virtually.”*

*(P014)*

*“We had some fun and game (umm) we had a terraced house (umm) they just about managed to get me in with a wheelchair.”*

*(P021)*

Being stuck in the home often led to feelings of social isolation.

*“While I was in this bungalow, this house. Very isolated, but… anyway I used to have to do my shopping on the internet to get anything because I couldn’t go anyway.”*

*(P008)*

#### 3.2.2. Adapting Behaviour

The nature of limb loss meant that individuals suddenly had to adapt their daily living, including the way that they moved around and used their own home. Individuals described the impact of this sudden change.

*“I basically learnt to adapt because it’s a case of all of a sudden ok I’m in the house, the old house…” *

*(P001)*

The participants described various way in which they had to adapt their own behaviour to enable them to function within their own home.

*“Not being able to move sideways, not being able to reach things on the shelves […] you know not being able to carry the kids (umm) upstairs or you know it’s a big palaver to be… you know to hold a child, to go into the lift, to go up in a lift to go through your bedroom to their bedroom, down steps. It was not all wheelchair friendly this house isn’t wheelchair friendly for me. It’s not… it’s not wheelchair for everybody.”*

*(P005)*

*“I was getting in the bath the wrong way, my leg was on the inside of the bath near the wall so… I couldn’t get out with my stump. So now I realise that I’ve got to turn around. Foot facing the taps, so this comes out first and then I’ve got the support and I just push up… and it’s so much easier.” *

*(P027)*

*“I clean the oven out, alright I don’t have my leg on because I sit on my bum doing it, but you normally anyway I mean because the ovens low down. So how else are you going to do it? You’re going to have to sit down or stoop down, but it’s stupid stooping down, you’ve got to sit down.”*

*(P012)*

The need to adapt behaviour was diverse in this population. For some, the complexity of the prosthesis, stump pain and multimorbidity impacted when they were able to use their prosthesis. This in turn impacted the way in which they were able to use their own home, and often meant that their needs were not consistent and constantly changed over time.

*“My [Genium X-5] (umm) but when (umm) when it’s not working properly and when I’m in spinal pain and pelvic hip pain, I can’t wear my leg so I have to go around on elbow crutches.” *

*(P002)*

*“And the deterioration side of things because of the accumulation of injuries and then the compounding effect of each against the other, means that maintaining mobility, independence is much more difficult than it was.”*

*(P009)*

## 4. Discussion

This study aimed to address the current gap in the field by examining the housing needs of veterans experiencing limb loss, and the impact of limb loss on housing needs and home adaptations across the life course of ageing military veterans. There were two overarching themes generated from the life-story interviews: availability of support and changing housing needs.

The results from this study demonstrated that participants often struggled to navigate the processes of acquiring financial support. For example, transitioning from service life brought significant structural barriers and led to inequity of access to healthcare. There are few, if any, jobs or careers outside the military that require as much adjustment as that of leaving the Armed Forces and returning to a civilian lifestyle. Veterans often find that their service life bears little or no resemblance to that experienced as a civilian member of society. Furthermore, wider research carried out with veterans who had experienced lower-limb amputation demonstrates that there are health literacy issues that impact on health-related quality of life [18]. The veterans’ transition review [19] recognises the influence of stereotyping and discrimination by employers towards veterans, such as preconceptions that they are institutionalised or aggressive. As such, veterans who experience limb loss are likely to encounter ‘double jeopardy’ because of limited employment opportunities and assumptions about capability that are made based on their disabilities. Securing appropriate housing or support for home adaptations from local authority, social care providers and third-sector housing organisations, facilitated a shift in direction of veterans’ stories while striving to maintain independence. In some cases, this was due to the sudden nature of limb loss and geographical relocation. Individuals were unsure who was responsible for this care and relied on military charities as a source of support to help navigate the system, or to provide their own source of financial support. Although circumstantially different, limited awareness of available funding, such as the DFG, and the options individuals have in modifying their home, is also common within the wider older population [20], prompting the need to raise awareness and widely promote this available support. Adams and Hodges [20] acknowledged the necessity of the breadth and consistency of information and advice services provided by local authorities across the UK, and recommend establishing a minimum standard. This research supports this need, and also the importance of recognising and supporting diverse needs and circumstances.

One source of provision available to military veterans that is inaccessible to the general population is the support from multiple military charities who offer housing support, housing maintenance and funding for home adaptations. The Royal British Legion offers financial advice on home adaptations [21] in assisting members of the Armed Forces Community to understand the financial support provided by local authorities and social care providers. In the first instance, the Royal British Legion state that they direct individuals to the Local Authority, but if an individual is ineligible they are redirected to the Independent Living service—a service which supports installation of small home adaptations, personal alarms or new equipment [21]. Similarly, volunteers at SSAFA (Soldiers, Sailors, Airmen and Families Association) provide guidance in accessing home adaptations and equipment. Blesma, the limbless veterans’ charity, provided grants for provision of wheelchairs, stair lifts and home and garden adaptations, as well as home and garden maintenance [22]. This online/telephone support was beneficial as one method of raising awareness to the statutory financial assistance that individuals may have been eligible for or, if eligibility for this financial assistance was not met, provided funding for home adaptations or modifications.

A further aspect which impacted access to home adaptations, although a characteristic that is not solely related to being in the military, is the stoic determination of participants to achieve success. The data suggests that this stoic attitude may prevent adaptive problem-orientation and the acceptance of home adaptations because it discourages emotional expressions and inhibits help-seeking behaviour. In addition, previous research has shown that military veterans can often set themselves apart from other disabled groups by identifying as ‘war heroes’. Veterans are known to be reluctant to engage in help-seeking behaviour, particularly when there is a perceived social stigma attached to those with those problems [18,23] and subsequently individuals can have quite varied levels of awareness of the support available [24]. The military context is important, as it links the ability to adjust to ‘disability’ with identity. This results in portrayal of personal disability due to limb loss as a symbol of courage.

Individuals described the multiple ways in which their home was no longer suitable, and they relied upon home adaptations to maintain daily living as well as modifying their own behaviour in order to be able to better manage their environment. Many participants did not use the prosthesis in their home as they felt that it restricted their movement. Some participants described using prosthetics intermittently because of pain and discomfort.

Following amputation, limb-fitting services, along with rehabilitation, are of significant importance in order to improve the prospect of maintaining independence. Resnik [25] suggests that the provision of suitable upper limb prostheses and rehabilitation services can improve satisfaction with a prosthetic limb itself and generally improve quality of life. However, some military veterans who have experienced upper limb amputation choose to abandon or refuse to use their prostheses because they are ill fitting and uncomfortable. It is imperative to consider the individuals’ own needs, and to understand that these needs may continuously adapt and evolve. Evidence within the field stresses the importance of personalisation, as modifications and adaptations are much more successful if an individual’s functional and emotional needs are considered, and when people are involved in the decision-making and installation processes [20,26]. Personalisation is necessary when considering home adaptations for individuals who have experienced limb loss, due to the intermittent use of their prostheses, pain and changing needs. Ongoing assessments would allow for understanding of an individual’s changing need.

This study has its limitations. It does not fully represent female veterans who have experienced limb loss as only two females took part in this study. Furthermore, all participants involved in this study were members of the limbless military charity, Blesma. Therefore, the findings are potentially not transferrable to non-members.

## 5. Conclusions

It is evident from this study that the housing needs of military veterans who have experienced limb loss are unique. The findings of this research reflect those of the Murrison Report [5] in that military veterans with limb loss are a unique service-user group in various ways: most specifically, in the notion of and nostalgia for their military culture, the challenges of transition to civilian life along with geographical relocation and the transfer of health and social care into local civilian services. This contextual complexity impacts individuals’ needs and access to required financial resources and services. However, in addition to the negative uniqueness, there are also positive uniqueness with the development of multiple parallel third sector services specifically for wounded, injured and sick veterans. Large UK military charities such as The Royal British Legion and Help for Heroes run specific veteran rehabilitation programmes that aim to address the unique challenges veterans with injuries face. Most importantly these are through life support services which all veterans will have access to. Arguably, the greatest challenge faced is the veterans’ own understanding and perception of the availability of support from the armed forces community and the NHS throughout their lifespan.

## Figures and Tables

**Table 1:** Mechanism and nature of participants’ limb loss (N = 32).

	Number	Percentage
**Mechanism of Limb Loss**		
*In-Service Attributable*	11	34.4
*In-Service Non-Attributable ^a^*	2	6.2
*In-Service Unclear*	1	3.1
*Post-Service Accident*	9	28.2
*Post-Service Attributable ^b^*	2	6.2
*Post-Service Illness*	7	21.9
**Nature of limb loss**		
*Above-knee*	14	43.8
*Below-Knee*	10	31.3
*Arm*	1	3.1
*Double amputee*	1	3.1
*Quadriplegic*	4	12.5
*Monoplegic*	1	3.1
*Through-knee*	1	3.1

^a^ Participants who lost their limb during service but not as a result of their military service. ^b^ Post-service attributable acknowledges those that lost their limb post-service as a direct result of their military service.

**Table 2:** Overarching themes and related sub-themes generated from the data.

Theme	Sub-Theme
Availability of support	Navigating sources of support
	Support from military charities
	Stoicism
Changing housing needs	Unsuitable housing
	Adapting behavior
